# Predicting Long-Term Mortality in Patients with Angina across the Spectrum of Dysglycemia: A Machine Learning Approach

**DOI:** 10.3390/diagnostics11061060

**Published:** 2021-06-09

**Authors:** Yu-Hsuan Li, Wayne Huey-Herng Sheu, Wen-Chao Yeh, Yung-Chun Chang, I-Te Lee

**Affiliations:** 1 Division of Endocrinology and Metabolism, Department of Internal Medicine, Taichung Veterans General Hospital, Taichung 40705, Taiwan; brightlight720720@gmail.com (Y.-H.L.); whhsheu@vghtc.gov.tw (W.H.-H.S.); 2 Department of Computer Science & Information Engineering, National Taiwan University, Taipei 10617, Taiwan; 3 School of Medicine, National Yang-Ming University, Taipei 11221, Taiwan; 4 Institute of Information Systems and Applications, National Tsing Hua University, Hsinchu 30013, Taiwan; s109065801@m109.nthu.edu.tw; 5 Graduate Institute of Data Science, Taipei Medical University, Taipei 10675, Taiwan; 6 Clinical Big Data Research Center, Taipei Medical University Hospital, Taipei 10675, Taiwan; 7 School of Medicine, Chung Shan Medical University, Taichung 40201, Taiwan; 8 College of Science, Tunghai University, Taichung 40704, Taiwan

**Keywords:** angiography, Brier score, Harrell’s C-index, least absolute shrinkage and selection operator, machine learning, oral glucose tolerance test

## Abstract

We aimed to develop and validate a model for predicting mortality in patients with angina across the spectrum of dysglycemia. A total of 1479 patients admitted for coronary angiography due to angina were enrolled. All-cause mortality served as the primary endpoint. The models were validated with five-fold cross validation to predict long-term mortality. The features selected by least absolute shrinkage and selection operator (LASSO) were age, heart rate, plasma glucose levels at 30 min and 120 min during an oral glucose tolerance test (OGTT), the use of angiotensin II receptor blockers, the use of diuretics, and smoking history. This best performing model was built using a random survival forest with selected features. It had a good discriminative ability (Harrell’s C-index: 0.829) and acceptable calibration (Brier score: 0.08) for predicting long-term mortality. Among patients with obstructive coronary artery disease confirmed by angiography, our model outperformed the Global Registry of Acute Coronary Events discharge score for mortality prediction (Harrell’s C-index: 0.829 vs. 0.739, *p* < 0.001). In conclusion, we developed a machine learning model to predict long-term mortality among patients with angina. With the integration of OGTT, the model could help to identify a high risk of mortality across the spectrum of dysglycemia.

## 1. Introduction

Angina is frequently encountered in patients with ischemic heart diseases [1]. Based on coronary angiography, ischemic heart disease can be classified as obstructive coronary artery disease (CAD) and non-obstructive CAD [1,2]. Obstructive CAD has an established association with increased cardiovascular mortality and is the target of treatment in contemporary practice [3,4]. However, a growing body of evidence has revealed that 50~70% of patients with angina have non-obstructive CAD on coronary angiography [5,6], and they are also associated with a high risk of all-cause mortality and cardiovascular events [7,8]. Since there are two entities of patients with angina, the European Society of Cardiology has published associated guidelines to address differences in their diagnosis and prognosis [9]. However, the optimal management strategy for patients with angina is still under debate, and a risk stratification model to identify high-risk patients and to tailor personal management is warranted [10,11,12].

Current risk stratification models for patients with ischemic heart disease are mainly based on patients with documented obstructive CAD, ranging from stable CAD to acute coronary syndrome [13]; however, a predictive model focused on patients with angina, including both obstructive and non-obstructive CAD, is still lacking. In addition, glucose perturbation represents a well-documented risk factor for atherosclerosis and is commonly seen in patients with ischemic heart disease [14]. A recent survey showed that dysglycemia detected by an oral glucose tolerance test (OGTT) is prevalent in patients with CAD [15]. However, contemporary predictive models for patients with ischemic heart disease seldom integrate glucose indices into their parameters. For example, the Global Registry of Acute Coronary Events (GRACE) discharge score [16], which is a widely used risk score for patients with acute coronary syndromes and has recently had its predictive ability validated for patients with CAD [17], has long been criticized for not including glucose indices [18]. Herein, we aimed to use data collected from patients with angina undergoing coronary angiography and OGTT to build a machine learning model to predict long-term mortality.

## 2. Materials and Methods

### 2.1. Setting and Participants

Data were obtained from a prospective, observational study conducted at Taichung Veterans General Hospital. The study enrolled adult patients admitted for coronary angiography between April 2009 and December 2018 due to symptoms of angina and under suspicion of ischemic heart disease by cardiologists. Patients were excluded if they had (a) a history of diabetes before this coronary angiography; (b) a fasting plasma glucose (FPG) ≥ 126 mg/dL during hospitalization; (c) a surgical history of coronary artery bypass graft; (d) acute or chronic infectious diseases; (e) severe systemic diseases, such as malignancies, autoimmune diseases, and psychiatric disorders; (f) an addiction to alcohol or drugs; or (g) pregnancy. All patients underwent OGTT after overnight fasting at an outpatient visit after discharge, and glucose levels were tested at fasting, at 30 min, and at 120 min during the OGTT. Normal glucose regulation was determined as the FPG < 100 mg/dL and the glucose level at 120 min (OGTT 120 min) was <140 mg/dL. Newly diagnosed diabetes was defined as the FPG ≥ 126 mg/dL or the OGTT 120 min ≥ 200 mg/dL. Prediabetes was defined in patients with a glucose regulation between that of normal glucose regulation and diabetes. Obstructive CAD was defined as at least a lesion with ≥50% stenosis, and non-obstructive CAD was defined as a lesion with <50% stenosis in coronary angiography reports. Baseline data collected upon admission, coronary angiography reports, OGTT results, and the medication at the outpatient visit were considered as candidate variables for analysis. Variables with >20% missing values were excluded, and multivariable imputation using the chained equation was applied for the remaining variables [19]. Mortality data up to December 2019 were retrieved from the Collaboration Center of Health Information Application, Department of Health, Executive Yuan, Taiwan, and served as the outcome of interest. The study complied with the Declaration of Helsinki and was approved by the Institutional Review Board of Taichung Veterans General Hospital. Written consent was obtained from each patient before the study procedures were performed (Trial Registration: NCT01198730, ClinicalTrials.gov, last accessed on 7 January 2020).

### 2.2. Model Development and Evaluation

Model development consisted of two parts. In the first part, we used all available variables to develop prediction models. We chose different machine learning methods, including random survival forest (RSF), gradient boosting machine learning algorithm (XGBoost) for survival analysis, and discrete-time survival model for neural networks [20,21]. RSF is an ensemble tree method for analysis of right censored survival data. It can handle complex interactions among variables, including mixed data types and nonlinear relationships between variables. XGBoost is a novel boosting tree-based ensemble algorithm whose performance is iteratively improved through the optimization of a customized objective function. To handle time-to-event data, the objective function of XGBoost was set as Cox regression. The discrete-time survival model for neural networks was implemented in the Keras deep learning framework and was trained with the maximum likelihood method using minibatch stochastic gradient descent. The likelihood function was used as the loss function, and it naturally incorporated non-proportional hazards. The prediction of these machine learning methods was the hazard ratio of each individual, and their results were compared with those of Cox proportional hazards models.

In the second part, we used features selected by the least absolute shrinkage and selection operator (LASSO)-derived Cox proportional hazards model to construct the predictive model. As the Cox proportional hazards model could not converge and face the risk of overfitting using all the variables those we collected, LASSO was introduced in the Cox model for regularization. LASSO regularization can shrink the estimations and force certain coefficients to zero, thereby keeping only the important features [22]. Significant predictors with *p* values < 0.05 in the LASSO-derived Cox proportional hazards model were selected as input variables for the machine learning methods mentioned above.

The prediction models were internally validated using a five-fold cross validation. Discrimination ability was assessed using the Harrell’s C-index [23]. Calibration was evaluated using the Brier score [5]. The Brier score measures the mean squared difference between the predicted probability and the actual outcome, with a lower score indicating better calibrated predictions. To explain the contribution of each variable to the best performing model, Shapley values were utilized [24,25]. Based on game theory, Shapley values can explain a model’s prediction by computing the importance of each feature to the prediction.

### 2.3. Comparison with GRACE Discharge Score

For patients with obstructive CAD confirmed by coronary angiography, we calculated their GRACE discharge score, the components of which included age, heart rate, systolic blood pressure, serum creatinine, the presence of congestive heart failure, cardiac arrest during admission, elevated cardiac enzyme, and ST segment deviation [16]. We compared the performance of GRACE with that of the best performing model. Net reclassification improvement (NRI) and integrated discrimination improvement (IDI) were used to evaluate the improvement in predictive power of our final model compared with the GRACE discharge score [26]. Analyses were performed using R 3.4 software (The R Project for Statistical Computing, Vienna, Austria) and Python (version 3.6).

## 3. Results

### 3.1. Characteristics of Enrolled Patients

A total of 1479 patients were included in the analyses, and 157 patients (10.6%) died during a median follow-up of 6 years (interquartile range, 3.1–9.1 years). Table 1 lists the cohort’s baseline characteristics. Briefly, patients who died tended to be older, with higher heart rates, higher urine albumin-to-creatinine ratios, higher uric acid levels, higher high-density lipoprotein cholesterol levels, higher FPG, and higher OGTT 120 min, whereas their body mass index, diastolic blood pressure, triglycerides, glutamic pyruvic transaminase, hemoglobulin, and estimated glomerular filtration rate were lower compared with patients who survived. Patients who died during follow-up had a lower prevalence of beta-blocker use and a higher prevalence of newly diagnosed diabetes, CAD history before admission, smoking history, and uses of angiotensin-converting enzyme inhibitors, angiotensin II receptor blockers (ARB), alpha blockers, and diuretics compared with patients who survived.

### 3.2. Feature Importance and Model Performance

**Figure 1:**
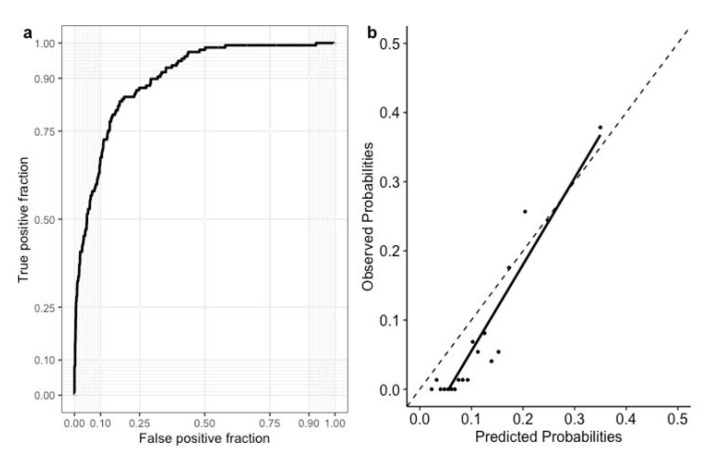
Area under the curve of the best performing model for predicting all-cause mortality among patients with angina (**a**). The closer to the dotted line indicates the better prediction in the calibration plot of the best performing model (**b**).

**Figure 2:**
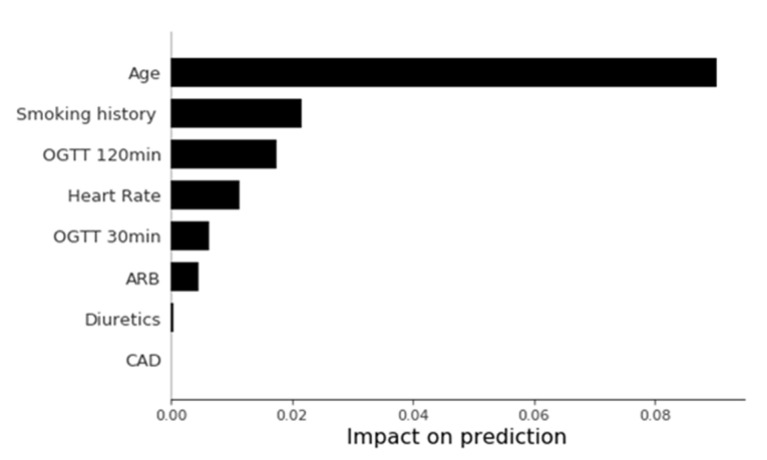
Impact of selected features on model prediction. The impact on prediction was based on the SHAP value, which was calculated from the best performing model. (ARB = angiotensin II receptor blocker, CAD = coronary artery disease, OGTT = oral glucose tolerance test, SHAP = Shapley Additive Explanation values).

The significant predictors selected by the LASSO-derived Cox proportional hazards model included age, heart rate, glucose level at 30 min (OGTT 30 min), OGTT 120 min, CAD history, smoking history, use of ARB, and use of diuretics (Table 2). The performance of the predictive models is shown in Table 3. The RSF model after feature selection had the highest Harrell’s C-index (0.829) and acceptable calibration (Brier score: 0.08). Figure 1 shows the area under the operating curve and the calibration plot of predicted risks at 10 years. Shapley values of the variables in the best performing model are shown in Figure 2.

### 3.3. Comparison with GRACE Discharge Score

Among patients with obstructive CAD confirmed by angiography during admission, Harrell’s C-index of the best performing model was significantly greater than the GRACE discharge score (0.829 vs. 0.739, respectively; *p* < 0.001). The NRI (0.328, 95% confidence interval [CI]: 0.096–0.583, *p* = 0.027) and IDI (0.135, 95% CI: 0.068–0.203, *p* = 0.007) indices also showed improvement in predictive ability compared with the GRACE discharge score model alone. Predictive ability was also significantly better in the best performing model than in the GRACE discharge score, with OGTT 120 min model. (Table 4).

## 4. Discussion

In this study, we built a machine learning-based model to predict long term mortality among patients with angina across the spectrum of dysglycemia. With glucose indices obtained from OGTT and other available clinical data, this model showed good discrimination and accuracy in predicting long-term mortality after coronary angiography. To the best of our knowledge, this study is among the first to compare state-of-the-art machine learning methods to predict survival in patients with angina, with an emphasis on OGTT results as important parameters.

For patients with obstructive CAD, several predictive models have been developed to predict major cardiovascular events and mortality. For example, the GRACE discharge score has been recently validated in its accuracy for predicting mortality 2 years after coronary angiography, with an area under the curve of 0.61 for patients with stable CAD [17]. The ABC-CHD score [27], with risk factors identified by the Cox proportional hazards model including age, biomarkers (N-terminal prohormone of brain natriuretic peptide and troponin-T), and clinical histories (smoking, diabetes, and presence of peripheral artery disease), has a good discriminatory ability (Harrell’s C-index: 0.71) and calibration for three-year mortality. However, previous models seldom include patients with non-obstructive CAD. It has been reported that more than half of patients with angina have no obstructive CAD during coronary angiography [28,29,30]. Since a sizable proportion of patients with angina has non-obstructive CAD, our model, which was derived from a cohort in which almost 50% of patients had non-obstructive CAD, is more representative of real-world patients with angina. Even for patients with obstructive CAD, our model outperformed the GRACE discharge score to predict long-term mortality.

The predictors detected by our LASSO-derived Cox proportional hazards models were age, diuretic use, ARB use, heart rate at admission, OGTT 120 min, and OGTT 30 min. Although some are well-established risk factors for mortality and have been included in previous predictive models for patients with CAD, using OGTT results as parameters for risk stratification has not been investigated before. Our study showed high accuracy in mortality prediction with the integration of OGTT results. According to the EUROASPIRE study, OGTT 120 min is a predictor of major cardiovascular events and mortality for patients without diabetes [31]. Chattopadhyay et al. [32] showed that, with the adjustment of OGTT 120 min, the GRACE discharge score has an improved prognostic ability for patients with acute coronary syndrome. Similar to above studies, our model containing OGTT 120 min outperformed the GRACE discharge score for predicting mortality among patients with angina, supporting the importance of OGTT 120 min for mortality prediction among patients with ischemic heart disease. According to our data at baseline, the mean FPG was in the normal range, but OGTT 120 min and glycated hemoglobin (HbA1c) were in the range of prediabetes. Postprandial hyperglycemia has been reported more in Asian patients in comparison to Caucasian patients [33,34]. Moreover, OGTT 120 min is more sensitive than FPG for diagnosing abnormal glucose regulation in patients with CAD [35], and is better than HbA1c and FPG for predicting major cardiovascular events [31].

Our model also revealed potentially unidentified predictors, such as OGTT 30 min. OGTT 30 min has predictive value for developing type 2 diabetes and is associated with inflammatory markers [36,37]; however, its role in mortality prediction has not been previously evaluated. Based on the Shapley value derived from our model, OGTT 30 min also contributed to mortality prediction in patients with angina. Further prospective studies are warranted to elucidate the prognostic value of OGTT 30 min. It is notable that several traditional cardiovascular risks were not selected for our predictive model. In this cohort which included patients with angina, all of the participants had undergone hospitalization where adequate medication and education were performed. Therefore, controlled cholesterol and blood pressure was observed in outpatient visits, and the attenuated contribution to traditional risk factors was close to that in real-world practice [38]. Moreover, the median duration between coronary angiography and outpatient visit was 13 days (interquartile range: 9–17 days) in the present study. Therefore, the low-density lipoprotein cholesterol might be not adequately decreased after a short-term treatment of statins.

There are several clinical applications of this model. Our model is derived from patients with angina. More than half of patients with angina have non-obstructive CAD, and approximately 20~40% of patients with CAD still suffer from angina symptoms after revascularization [4]. There is heterogeneity in prognosis among patients with angina, and it is important to stratify their risk and tailor their management strategy. However, contemporary clinical practice mainly focuses on the prevention and management of obstructive CAD [39], despite the fact that the risk of major cardiovascular events and mortality among patients with non-obstructive CAD is increased [40]. Our model could help to identify patients with angina at a high risk of mortality. In addition, our model emphasized the importance of OGTT. Screening for dysglycemia using OGTT in patients undergoing percutaneous coronary intervention has long been proposed and is also recommended in European Society of Cardiology guidelines [40,41]. However, adhesion to this recommendation is poor [35], partially because the prognostic role of OGTT is less clear. Although early intervention using anti-diabetic drugs did not significantly reduce the risk of a major adverse cardiovascular event in the Acarbose Cardiovascular Evaluation trial [42], other intensive treatment, e.g., high-intensity statins, is highly recommended for patients with high-event risk [43]. Our model, which adds prognostic value to the OGTT for patients with angina, could increase adherence to this recommendation.

The major strength of this study is that we used the LASSO-derived Cox proportional hazards model for feature selection and advanced machine learning methods for model development. Only six variables were needed in our model after utilization of LASSO regularization, and most of them, except OGTT, were available from electronic health records, making it a convenient tool to implement in clinical practice. The best performing machine method in our study was RSF. Previous predictive models for CAD were usually built using Cox proportional hazards models; however, several assumptions must be met before applying the Cox proportional hazards model. Conversely, machine learning methods, such as RSF, can handle non-linear, complex relationships between features without assumptions, thus widening their clinical application. However, there are still some limitations which should be highlighted in the current study. First, coronary angiography was arranged by cardiologists according to clinical judgement, including inconclusive non-invasive tests, unresponsive to medical therapies, or risks of high event. However, we did not examine the microvascular dysfunction using invasive stress tests in the enrolled patients without significant coronary obstruction [9]. Second, we did not follow the changes in medical treatment after baseline assessments. Third, this cohort has been active since 2009, and contemporary anti-diabetic medications, such as sodium glucose co-transporters 2 inhibitors and glucagon-like peptide-1 receptor agonists, which can reduce mortality risk in patients with type 2 diabetes, were seldom prescribed. Finally, our model has not been externally validated with other independent datasets in the present study, so its performance in other datasets is unknown. Further external validation using an independent dataset is scheduled.

## 5. Conclusions

We developed a machine learning model containing OGTT results and other clinically available parameters to predict all-cause mortality among patients with angina. This model could help to identify patients at a high risk of mortality. With the integration of glucose indices from OGTT, patients with dysglycemia could be identified early, enabling their risk of mortality to be accurately evaluated.

## Figures and Tables

**Table 1:** Baseline characteristics of the study population.

	Survival (N = 1322)	Death (N = 157)	*p*
Age (years)	58 ± 10	72 ± 12	<0.001
Female (n, %)	238 (18%)	20 (13%)	0.122
Body height (cm)	165 ± 8	162 ± 8	<0.001
Body weight (kg)	71.3 ± 11.7	66.4 ± 12.2	<0.001
BMI (kg/m^2^)	26.1 ± 3.4	25.2 ± 3.9	0.004
Waist (cm)	90.6 ± 8	90.5 ± 9.4	0.923
SBP (mmHg)	126 ± 17	128 ± 20	0.226
DBP (mmHg)	74 ± 10	69 ± 11	<0.001
Heart rate (beat/min)	71 ± 11	75 ± 13	<0.001
UACR (mg/g)	36 ± 176	111 ± 274	<0.001
Uric acid (mg/dL)	6.6 ± 1.6	7.3 ± 2.0	<0.001
Triglycerides (mg/dL)	147 ± 105	122 ± 78	0.004
Total cholesterol (mg/dL)	173 ± 39	169 ± 35	0.253
HDL cholesterol (mg/dL)	46 ± 12	49 ± 15	0.007
LDL cholesterol (mg/dL)	106 ± 34	103 ± 31	0.501
Creatinine (mg/dL)	1.06 ± 1.01	1.01 ±0.57	0.298
eGFR (mL/min/1.73 m^2^)	82 ± 22	63 ± 21	<0.001
GPT (U/L)	32 ± 32	26 ± 19	0.028
Hemoglobin (g/dL)	13.9 ± 1.5	13.2 ± 1.9	0.001
WBC (/μL)	6898 ± 2772	6923 ± 2176	0.914
CK (U/L)	151 ± 314	180 ± 370	0.373
CKMB (U/L)	9 ± 16	11 ± 16	0.241
Troponin-T (ng/L)	3.9 ± 19.5	1.4 ± 3.6	0.209
OGTT (mg/dL)			
Glucose 0 min	95 ± 14	100 ± 19	<0.001
Glucose 30 min	169 ± 32	169 ± 36	0.962
Glucose 120 min	145 ± 50	166 ± 59	<0.001
HbA1c (%)	5.8 ± 0.6	6.1 ± 0.8	<0.001
Glucose status (n, %)			<0.001
Normal glucose regulation	428 (32.4%)	43 (27.4%)	
Prediabetes	637 (48.2%)	51 (32.5%)	
Diabetes	257 (19.4%)	63 (40.1%)	
Smoking status (n, %)			<0.001
Non-smoker	590 (44.6%)	52 (33.1%)	
Smoker	318 (24.1%)	18 (11.5%)	
Ex-smoker	414 (31.3%)	87 (55.4%)	
Medication (n, %)			
Antiplatelet	1226 (92.8%)	147 (93.6%)	0.830
ACE inhibitor	278 (21.0%)	46 (29.3%)	0.023
ARB	415 (31.4%)	69 (43.9%)	0.002
Alpha blocker	54 (4.1%)	16 (10.2%)	0.001
Beta blocker	368 (27.8%)	23 (14.6%)	0.001
CCB	689 (52.1%)	78 (49.7%)	0.622
Diuretics	153 (11.6%)	44 (28.0%)	<0.001
CAD history * (n, %)	140 (10.6%)	26 (16.6%)	0.035
Grace score	90.2±20.3	125.3 ± 31	<0.001
Left ventricular ejection fraction (%)	52 ± 11	47 ± 13	<0.001
Number of coronary arteries with significant stenosis ^†^ (n, %)			0.007
Non-obstructive CAD	611 (46.2%)	51 (32.5%)	
1	356 (26.9%)	48 (30.6%)	
2	245 (18.5%)	42 (26.8%)	
3	110 (8.3%)	16 (10.2%)	
Non-invasive studies before angiography (n, %)			
Treadmill exercise test	575 (43.5%)	66 (42.0%)	0.793
Myocardial perfusion imaging	116 (8.8%)	16 (10.2%)	0.660
Echocardiography	282 (21.3%)	30 (19.1%)	0.588
Rest electrocardiography	349 (26.4%)	45 (28.7%)	0.609
Percutaneous coronary intervention (n, %)			
without stent insertion	146 (11.0%)	36 (22.9%)	<0.001
with stent insertion	542 (41.0%)	67 (42.7%)	0.640

* CAD history refers to percutaneous coronary intervention for obstructive CAD before admission; ^†^ Significant stenosis defined as stenosis ≥ 50%. ACE = angiotensin-converting enzyme, ARB = angiotensin II receptor blocker, BMI = body mass index, BP = blood pressure, eGFR = estimated glomerular filtration rate, FPG = fasting plasma glucose, HDL = high-density lipoprotein, LDL = low-density lipoprotein, OGTT = oral glucose tolerance test, SBP = systolic blood pressure, DBP = diastolic blood pressure, CCB = calcium channel blocker, CAD = coronary artery disease, WBC = white blood cell count, CK = creatine kinase, GPT = glutamic pyruvic transaminase, HbA1c = glycated hemoglobin, UACR = albumin-to-creatinine ratio.

**Table 2:** Features selected by the LASSO-derived Cox proportional hazards model.

	HR	95% CI	*p*
Age	1.06	(1.04–0.08)	<0.001
Heart rate	1.02	(1.01–1.04)	<0.001
OGTT 30 min	0.98	(0.97–0.99)	<0.001
OGTT 120 min	1.01	(1.01–1.02)	<0.001
CAD history	1.66	(1.03–2.66)	0.040
Smoking history	1.31	(1.06–1.65)	0.013
ARB use	1.74	(1.18–2.55)	0.008
Diuretic use	1.57	(1.01–2.38)	0.048

ARB = angiotensin II receptor blocker, CAD = coronary artery disease, CI = confidence interval, HR = hazard ratio, LASSO = least absolute shrinkage and selection operator, OGTT = oral glucose tolerance test.

**Table 3:** Performance of different machine learning methods.

	Harrell’s C-Index	Brier Score
With all variables		
Cox regression	0.774	0.069
RSF	0.804	0.082
XGBoost	0.788	0.033
DNN	0.750	0.102
With selected features		
Cox regression	0.741	0.076
RSF	0.829	0.080
XGBoost	0.794	0.056
DNN	0.796	0.106

RSF = random survival forest, XGBoost = extreme gradient boosting, DNN = dense neural network.

**Table 4:** GRACE-score-based models compared to the best-performing model.

Model	Harrell’s C-Index (95% CI)	*p*	Absolute IDI (95% CI)	*p*	NRI (95% CI)	*p*
GRACE score	0.739 (0.683, 0.796)	<0.001	0.135 (0.068, 0.203)	0.007	0.328 (0.096, 0.583)	0.027
GRACE score + OGTT 120 min	0.740 (0.685, 0.797)	<0.001	0.115 (0.033, 0.224)	0.027	0.336 (0.103, 0.646)	0.027
RSF	0.829 (0.790, 0.869)					

CI = confidence interval; GRACE = Global Registry of Acute Coronary Events; IDI = integrated discrimination improvement; NRI = net reclassification improvement; OGTT = oral glucose tolerance test; RSF = random survival forest with selected features.

## Data Availability

Data available on request due to privacy.
